# Continuous High-Precision Positioning in Smartphones by FGO-Based Fusion of GNSS–PPK and PDR

**DOI:** 10.3390/mi15091141

**Published:** 2024-09-11

**Authors:** Amjad Hussain Magsi, Luis Enrique Díez, Stefan Knauth

**Affiliations:** 1 Faculty of Engineering, University of Deusto, Avda. Universidades 24, 48007 Bilbao, Spain; luis.enrique.diez@deusto.es; 2 Hochschule für Technik Stuttgart, Faculty of Computer Science, Geomatics and Mathematics, Schellingstraße 24, 70174 Stuttgart, Germany

**Keywords:** factor graph optimization, fusion, GNSS, Kalman Filter, PDR, post-processing kinematics, smartphone

## Abstract

The availability of raw Global Navigation Satellites System (GNSS) measurements in Android smartphones fosters advancements in high-precision positioning for mass-market devices. However, challenges like inconsistent pseudo-range and carrier phase observations, limited dual-frequency data integrity, and unidentified hardware biases on the receiver side prevent the ambiguity resolution of smartphone GNSS. Consequently, relying solely on GNSS for high-precision positioning may result in frequent cycle slips in complex conditions such as deep urban canyons, underpasses, forests, and indoor areas due to non-line-of-sight (NLOS) and multipath conditions. Inertial/GNSS fusion is the traditional common solution to tackle these challenges because of their complementary capabilities. For pedestrians and smartphones with low-cost inertial sensors, the usual architecture is Pedestrian Dead Reckoning (PDR)+ GNSS. In addition to this, different GNSS processing techniques like Precise Point Positioning (PPP) and Real-Time Kinematic (RTK) have also been integrated with INS. However, integration with PDR has been limited and only with Kalman Filter (KF) and its variants being the main fusion techniques. Recently, Factor Graph Optimization (FGO) has started to be used as a fusion technique due to its superior accuracy. To the best of our knowledge, on the one hand, no work has tested the fusion of GNSS Post-Processed Kinematics (PPK) and PDR on smartphones. And, on the other hand, the works that have evaluated the fusion of GNSS and PDR employing FGO have always performed it using the GNSS Single-Point Positioning (SPP) technique. Therefore, this work aims to combine the use of the GNSS PPK technique and the FGO fusion technique to evaluate the improvement in accuracy that can be obtained on a smartphone compared with the usual GNSS SPP and KF fusion strategies. We improved the Google Pixel 4 smartphone GNSS using Post-Processed Kinematics (PPK) with the open-source RTKLIB 2.4.3 software, then fused it with PDR via KF and FGO for comparison in offline mode. Our findings indicate that FGO-based PDR+GNSS–PPK improves accuracy by 22.5% compared with FGO-based PDR+GNSS–SPP, which shows smartphones obtain high-precision positioning with the implementation of GNSS–PPK via FGO.

## 1. Introduction

The Global Navigation Satellite System (GNSS) provides Positioning, Navigation, and Timing (PNT) services across diverse fields such as transportation, autonomous vehicles, disaster forecasting, aerospace, and defense. Since these services have traditionally relied on specialized expertise, costly and high-precision professional GNSS receivers. Therefore, their widespread use has been limited. However, nowadays, chip-size GNSS receivers have empowered devices like smartphones, tablets, smartwatches, and other portable gadgets to provide positioning services broadly. The use of smartphones for pedestrian positioning and navigation has become more widespread due to their mass availability and ease of access for individuals [1,2].

Before 2016, Android smartphones only provided position and velocity data, limiting research on smartphone-based positioning [3]. In May 2016, Google enabled access to raw GNSS data via the Android API, allowing for advancements in high-precision positioning. However, access to raw GNSS observations from smartphones does not solve the problem of high-accuracy localization systems via smartphones, as they are typically equipped with low-cost, poorly performed polarized GNSS antennas and unknown hardware biases on the receiver, resulting in noisy data and frequent cycle slips, especially in GNSS-challenging areas such as urban canyons, complex environments, tunnels, underpasses, etc., where these issues typically arise due to multipath interference and non-line-of-sight (NLOS) conditions [4,5]. Additionally, PNT signals from satellites are considerably impacted by various errors stemming from atmospheric conditions, including ionospheric delays, dry and wet tropospheric delays, as well as satellite clock offsets and ephemeris inaccuracies [6]. Therefore, the All Source Positioning and Navigation (APNS) program under the USA-based Defense Advanced Research Projects Agency (DARPA) has noted that single-source navigation is no longer reliable, prompting researchers to explore alternative navigation solutions [7,8]. This led researchers to fuse alternative navigation solutions to mitigate these problems. Different sensors are already available in modern smartphones, but among these, the Microelectrical Mechanical Systems (MEMS) Inertial Measurement Unit (IMU) holds significant importance due to its complementary behavior with GNSS, offering low errors in the short term and a high update rate without requiring additional infrastructure in the environment [9]. MEMS IMUs face limitations due to their high noise output, preventing the development of Strapdown Inertial Navigation Systems (SINS) and their integration with GNSS. This accumulates errors that are increased with the elapsed time, while the PDR system accumulates errors based on the detected steps, as they rely on pedestrian walking patterns [10]. Therefore, for the pedestrian navigation context with smartphones, PDR+GNSS fusion is preferable.

**Figure 1:**
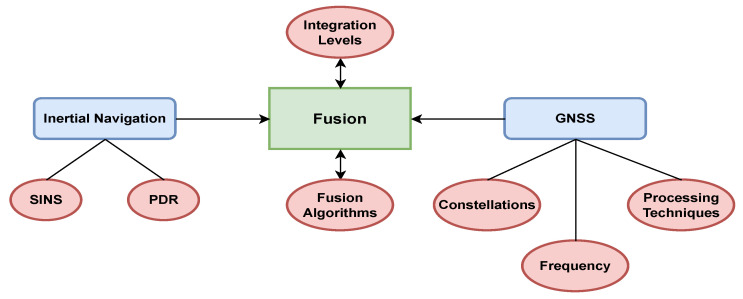
Inertial+GNSS fusion architectures.

The fusion of PDR and GNSS in smartphones is still an open research topic. These fusion architectures mainly involve several steps, such as integration levels, fusion algorithms and frequencies, constellation, and GNSS processing techniques, as shown in Figure 1. Most of the researchers used KF and its variant models as the main fusion technique for these fusion architectures, such as Jiang et al. [11], who used the Krill Herd Algorithm (KHA) to optimize particle filters for GNSS/PDR integration, enhancing positioning accuracy. Zhu et al. [12] improved GNSS/PDR fusion positioning based on smartphone IMU error analysis. Zhang et al. [13] assessed GNSS/PDR fusion on smartphones in complex settings. Yan et al. [14] modified the KF using varied sampling frequencies from smartphone GNSS and IMU data for fusion. However, achieving better accuracy with a KF is challenging due to its first-order Markov assumption, Gaussian noise assumption, and reliance on the most recent observation.

Recently, FGO-based multi-sensor fusion has gained attraction among researchers. Initially, FGO demonstrated superior accuracy in SLAM-based complex problems in robotics and has been applied to INS/GNSS fusion architectures as well [15,16,17,18,19,20,21,22]. However, in the pedestrian navigation domain, significant analysis is still needed, and the application of FGO is in its early stages, presenting open research opportunities. Recently, Jiang et al. [23] implemented FGO for PDR+GNSS for a single walking dataset in open-sky conditions and compared its performance against KF. Later, ref. [24] introduced a PDR/GNSS integration method using FGO to boost accuracy in challenging GNSS conditions. Later, ref. [25] tested FGO for PDR/GNSS on smartphones by placing the smartphone in different positions, such as box, handheld, and pocket, and tested the fusion performance with open sky and urban canyon conditions. In our previous paper [26], we compared the performance of FGO and KF under two different motion scenarios (walking and running) in partially open sky conditions. We found that FGO performance deteriorated with varying motions, with running significantly affecting FGO performance more than walking. This degradation was attributed to differences in motion parameters such as step length, stride length, and speed. Therefore, in another paper [27], we conducted a more detailed analysis of FGO by examining a range of pedestrian motions from different individuals, including variations in gender, gait patterns, and heights. Additionally, we investigated how transient variations in how the smartphone is held affect errors in the PDR block. We then evaluated FGO’s performance against these errors and compared it with KF and Smooth KF under these sources of PDR errors.

To the best of our knowledge, on the one hand, no work has tested the fusion of GNSS PPK and PDR on smartphones. And, on the other hand, the works that have evaluated the fusion of GNSS and PDR by means of FGO have always performed it using the GNSS SPP technique. Therefore, the aim of this work is to combine the use of the GNSS PPK technique and the FGO fusion technique to evaluate the improvement in accuracy that can be obtained on a smartphone compared with the usual GNSS SPP and KF fusion strategies.

The remaining sections detail the methodology, covering the PDR development and PDR+GNSS (SPP/PPK) fusion architectures via KF and FGO. The experimental evaluation section outlines the experiment’s description, setup, and results, followed by the conclusion and future work section.

## 2. Methodology

The PDR development and its fusion with GNSS–SPP and GNSS–PPK by KF and FGO are presented in this section.

### 2.1. PDR

**Figure 2:**
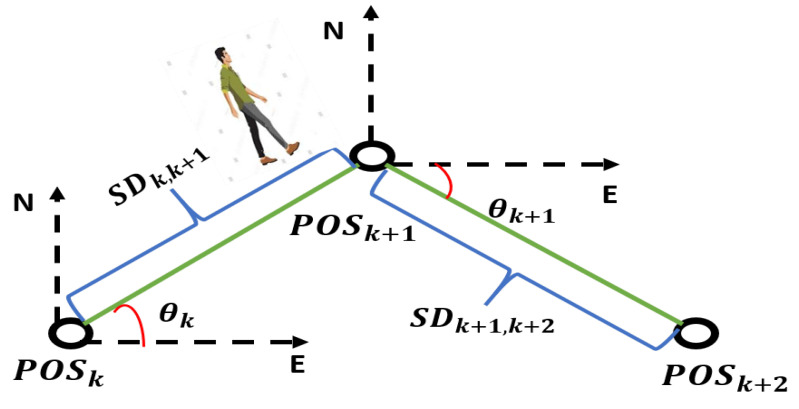
PDR mechanism.

By using the number of detected steps, step length, and heading estimation calculation, the PDR can be developed, and its mechanism shown in Figure 2 can be mathematically written as follows:

(1) $${\mathrm{Pos}}_{k+1}={\mathrm{Pos}}_{k}+\Delta {\mathrm{Pos}}_{k|k+1}$$

In this equation, ${\mathrm{Pos}}_{k+1}$ and ${\mathrm{Pos}}_{k}$ show position at steps k+1 and *k*, respectively, and $\Delta {\mathrm{Pos}}_{k|k+1}$ is a positional increment from *k* to k+1, which can be formulated as follows in east and north directions.

(2) $$\Delta {\mathrm{Pos}}_{k|k+1}=\left[\begin{array}{c} \Delta {\mathrm{Pos}}_{k|k+1}^{E}\\ \Delta {\mathrm{Pos}}_{k|k+1}^{N} \end{array}\right]=\left[\begin{array}{c} {\mathrm{SL}}_{k|k+1}\cdot \cos\left(\phi \right)\\ {\mathrm{SL}}_{k|k+1}\cdot \sin\left(\phi \right) \end{array}\right]$$

ϕ is the heading angle, and ${\mathrm{SL}}_{k|k+1}$ is the step length between two steps.

The steps taken were detected by measuring the peak of acceleration magnitude, which can be written as

(3) $${\mathrm{Acc}}_{mag_{k}}=\sqrt{{\mathrm{Acc}}_{x_{k}}^{2}+{\mathrm{Acc}}_{y_{k}}^{2}+{\mathrm{Acc}}_{z_{k}}^{2}}$$

where ${\mathrm{Acc}}_{mag_{k}}$ denotes the magnitude of the triaxial accelerometer, and ${\mathrm{Acc}}_{x_{k}}$, ${\mathrm{Acc}}_{y_{k}}$, and ${\mathrm{Acc}}_{z_{k}}$ denote the accelerometer measurements in *x*, *y*, and *z* directions at time step *k*.

The step length was estimated using Weinberg’s algorithm [28], which can be mathematically formulated as follows:

(4) $${\mathrm{SL}}_{k|k+1}=C\cdot {\left[\max\left({\mathrm{Acc}}_{mag_{k}}\right)-\min\left({\mathrm{Acc}}_{mag_{k}}\right)\right]}^{1/4}\;$$

${\mathrm{SL}}_{k|k+1}$ denotes the estimated step length between two successive acceleration peaks, while *C* is a constant factor to adjust the estimated step length, and the step heading was estimated via the Attitude and Heading Reference System (AHRS) from the smartphone. The factor value of *C* was determined through empirical testing. We conducted experiments by adjusting *C* under various conditions to see how it influenced the accuracy and consistency of the results. Through this process, we identified a value for *C* that yielded the best performance for our specific application.

### 2.2. GNSS

Modern Android smartphones can now provide raw GNSS observations, including pseudorange measurements, carrier phase data, and navigation messages. This capability allows for high-accuracy positioning directly on smartphones by processing these raw data to minimize associated errors [29,30,31]. In this study, we have utilized Post-Processing Kinematic (PPK) and compared it with Single-Point Positioning (SPP). A brief description of these techniques is provided below.

Single-Point Positioning (SPP): Also known as standalone positioning, it is a straightforward method that relies solely on the code–phase pseudorange measurements to determine a receiver’s position. This method does not incorporate the more precise carrier phase measurements and typically does not apply error corrections [32,33]. The basic pseudorange equation used in SPP is as follows:

(5) $$\rho_{i}=c\left(t_{r}-t_{s}\right)+d_{orb}+d_{iono}+d_{trop}+\epsilon$$

where

- $\rho_{i}$ is the pseudorange from satellite *i*;
- *c* is the speed of light;
- $t_{r}$ and $t_{s}$ are the receiver and satellite clock biases, respectively;
- $d_{orb}$ represents satellite orbit errors;
- $d_{iono}$ and $d_{trop}$ are the ionospheric and tropospheric delays;
- ϵ encompasses various noise sources and multipath errors.

In SPP, the receiver calculates its position by solving for the location using pseudorange data from multiple satellites. However, due to the nature of code–phase measurements, this method is subject to several sources of error:

- **Clock offsets:** the receiver’s clock is less accurate compared with the atomic clocks on GNSS satellites, leading to timing errors.
- **Ephemeris errors:** inaccuracies in the satellite’s transmitted position.
- **Ionospheric and tropospheric delays:** signal propagation is affected by the Earth’s atmosphere, causing delays.
- **Multipath and NLOS (non-line-of-sight):** signals reflecting off surfaces before reaching the receiver, leading to errors in the measured pseudorange.

Because SPP does not correct for these errors and relies only on code data, the resulting position estimates are typically less accurate, often with errors in the range of several meters.

**Post-Processed Kinematics (PPK):** In contrast, PPK positioning is a more sophisticated method that uses carrier phase measurements, which are significantly more accurate than code–phase measurements. PPK applies a series of corrections to the raw GNSS data to achieve better accuracy, which is basically a post-processed RTK GNSS processing technique [34,35]. We opted for this approach because the SAPOS base station services in Germany did not provide real-time correction data. Instead, we had to download the RINEX observation files from the base station and process them afterward using RTKLIB Explorer. Additionally, we applied several other corrections, including ionospheric and tropospheric delay corrections, ambiguity fixing ratios, and adjustments to elevation angles. The basic equation for carrier phase measurement is as follows:

(6) $$\Phi_{i}=\rho_{i}+\lambda N_{i}+c\left(t_{r}-t_{s}\right)+d_{orb}+d_{iono}+d_{trop}+\epsilon_{\Phi }$$

where

- $\Phi_{i}$ is the carrier phase measurement;
- λ is the carrier wavelength;
- $N_{i}$ is the integer ambiguity (number of whole carrier wavelengths between the satellite and the receiver);
- Other terms are similar to those in the pseudorange equation, with $\epsilon_{\Phi }$ representing the measurement noise specific to carrier phase.

Key aspects of PPK include the following:

- **Ambiguity resolution:** PPK involves resolving the integer ambiguity $N_{i}$, which is crucial for achieving high precision. This often requires a reference station (a nearby base station) with known coordinates to help resolve these ambiguities.
- **Error corrections:** PPK applies corrections for ionospheric and tropospheric delays, satellite clock errors, and ephemeris errors. These corrections are typically derived from precise models or additional reference stations.

By correcting for these errors and resolving ambiguities, PPK can achieve positioning accuracy down much better than SPP.

### 2.3. PDR Fusion with GNSS–SPP and PPK with KF in Smartphones

The fusion of PDR and GNSS (both SPP and PPK) using KF has been performed following an error state approach and a loosely coupled architecture. X can be the state vector for transitioning the state addressing position errors in both the *x* and *y* coordinates of PDR and can be written as follows:

$$X=\left(\begin{array}{c} \Delta {\mathrm{PDR}}_{x}\\ \Delta {\mathrm{PDR}}_{y} \end{array}\right)$$

The formulation of this transformation further can be written as below:

(7) $$X_{k+1}=F_{k|k+1}\cdot X_{k}+W_{k+1}$$

In this equation, $X_{k+1}$ and $X_{k}$ are the state vectors. $W_{k+1}$ is a process noise vector with the assumption of normal Gaussian distribution as $W_{k+1}∼N\left(0,Q_{k}\right)$, where $Q_{k}$ is the covariance matrix of the process noise and $F_{k|k+1}$ is the transformation matrix at epochs k+1 and *k*, which can be expressed mathematically as

(8) $$F_{k|k+1}=\left[\begin{array}{cc} 1 & 0\\ 0 & 1 \end{array}\right]$$

The measurement model is

(9) $$Z_{k+1}=H_{k+1}\cdot X_{k+1}+V_{k+1}$$

where $Z_{k+1}$ represents the measurement vector. $V_{k+1}$ is the measurement noise vector with Gaussian distribution assumption expressed as $V_{k+1}∼N\left(0,R\right)$, where *R* represents the covariance matrix of the measurement noise and $H_{k+1}$ represents the observation matrix.

(10) $$H_{k+1}=\left[\begin{array}{cc} 1 & 0\\ 0 & 1 \end{array}\right]$$

The real observations represent the difference between PDR and GNSS–SPP for PDR+GNSS–SPP and PDR and GNSS–PPK for PDR+GNSS–PPK. Therefore, it can be rewritten and described as

(11) $$Z_{k+1}={\mathrm{Pos}}_{k+1}^{GNSS}-{\mathrm{Pos}}_{k+1}^{PDR}$$

Prediction from KF will be as follows:

(12) $$X_{k+1}^{-}=F_{k|k+1}\cdot X_{k}$$

(13) $$P_{k+1}^{-}=F_{k|k+1}\cdot P_{k}\cdot {\left(F_{k|k+1}\right)}^{T}+Q_{k}$$

The prediction state $X_{k+1}^{-}$ is derived via the transformation model. The matrix $P_{k}$ denotes the predicted state covariance matrix at epoch *k*, while $P_{k+1}^{-}$ represents the prediction at epoch k+1. Subsequently, the correction step of the Kalman filter updates the state estimation as follows:

(14) $${\hat{X}}_{k+1}=X_{k+1}^{-}+K_{k+1}\cdot \left(Z_{k+1}-H_{k+1}\cdot X_{k+1}^{-}\right)$$

(15) $$K_{k+1}=P_{k+1}^{-}\cdot {\left(H_{k+1}\right)}^{T}\cdot {\left[H_{k+1}\cdot P_{k+1}^{-}\cdot {\left(H_{k+1}\right)}^{T}+R_{k+1}\right]}^{-1}$$

(16) $$P_{k+1}=\left(I_{2\times 2}-K_{k+1}\cdot H_{k+1}\right)\cdot P_{k+1}^{-}$$

Here, ${\hat{X}}_{k+1}$ denotes the revised state estimation at time step k+1, $K_{k+1}$ symbolizes the Kalman gain matrix at time step k+1, and $I_{2\times 2}$ denotes the 2 × 2 identity matrix.

### 2.4. PDR Fusion with GNSS–SPP and GNSS–PPK with FGO in Smartphones

A factor graph serves as a probabilistic model that employs a graph structure to illustrate the probabilistic relationships among various variables. In essence, it consists of two distinct types of nodes: the unknown state variables and the factor nodes, which encode the conditional probabilities associated with certain sets of state variables. These nodes are connected by undirected edges, linking each state variable node to its corresponding factor node. An edge exists between these nodes only if there’s a functional dependence between the variable and factor. By employing a factor graph, one can break down a global probability function into the product of several local probability functions, thereby simplifying the resolution of the overall function.

In sensor integration, our focus typically lies on estimating a collection of state variables *X* based on a given set of measurements *Z*. Under the assumption of a first-order Markov model, we can express the conditional probability density function in the following manner:

(17) $$P\left(X|Z\right)=\prod_{i=1}^{k}\frac{P\left(z_{i}|x_{i}\right)P\left(x_{i}|x_{i-1},u_{i}\right)}{P(z_{i})}P\left(x_{0}\right)$$

where $z_{i}$ represents the measurements observed at epoch i (e.g., GNSS measurements), $x_{i}$ represents the system state at *i*, and $u_{i}$ denotes the control input (e.g., PDR measurements).

The most often used estimator for these unknown state variables X is the maximum a posteriori, or MAP estimate:

(18) $$\hat{X}=\arg\;\max P\left(X|Z\right)=\arg\;\max\prod_{i=1}^{k}P\left(z_{i}|x_{i}\right)P\left(x_{i}|x_{i-1},u_{i}\right)$$

In FGO-based integration, all these likelihood and transition probabilities are treated as the factorization of the global probability:

(19) $$\hat{X}=\arg\;\max\prod_{j=1}^{n}f\left(x_{j}\right)$$

Each factor models a constraint and must include a measure of uncertainty. The most common model is a Gaussian noise:

(20) $$P\left(z_{i}|x_{i}\right)\propto \exp\left(-\frac{1}{2}{∥h_{i}\left(x_{i}-z_{i}\right)∥}^{2}{▿}_{i}\right)$$

(21) $$P\left(xi|x_{i-1}\right)\propto \exp\left(-\frac{1}{2}{∥\Phi_{i}\left(x_{i-1}-x_{i}\right)∥}^{2}\Omega_{i}\right)$$

where the function $\Phi_{i}\left(\cdot \right)$ describes the relationship between the preceding states $x_{i-1}$ and $x_{i}$, the function $h_{i}\left(\cdot \right)$ represents the relationship between the state $x_{i}$ and the measurement $z_{i}$, and the covariance matrices are denoted by ${▿}_{i}$ and $\Omega_{i}$.

Taking the negative log and dropping the factor 1/2 transforms the problem into the minimization of an error function, which is a nonlinear least-squares problem.

(22) $$\hat{X}=\arg\min\left(\sum_{i=1}^{k}{∥\Phi_{i}\left(x_{i-1}\right)-x_{i}∥}_{\Omega_{i}}^{2}+\sum_{i=1}^{k}{∥h_{i}\left(x_{i}\right)-z_{i}∥}_{{▿}_{i}}^{2}\right)$$

**Figure 3:**
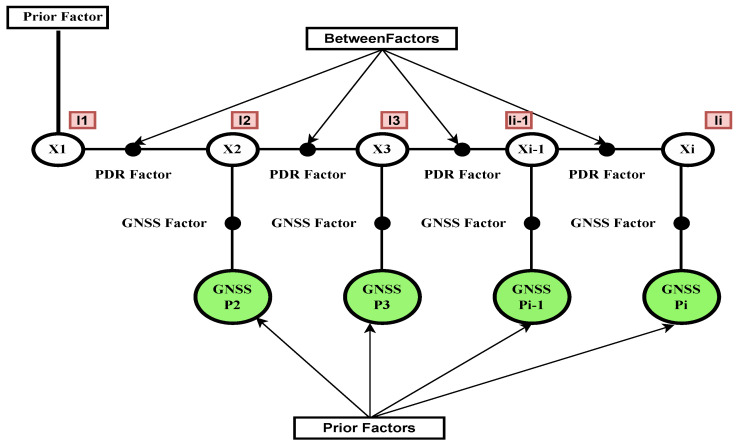
Factor graph for PDR+GNSS–SPP fusion architecture.

In our case, there are two factors: PDR measurements and the GNSS measurements (GNSS–SPP in case of PDR+GNSS–SPP) and (GNSS–PPK in case of PDR+GNSS–PPK) fusion architectures as shown in Figure 3.

**Factor by PDR position:** this factor is obtained from the positions derived by the PDR block.

(23) $$\begin{array}{c} {∥e_{k+1}^{PDR}∥}_{\sum_{k+1}^{PDR}}^{2}={∥X_{k+1}-f\left(X_{k}\right)∥}_{\sum_{k+1}^{PDR}}^{2}\\ ={∥x_{k+1}-\left(x_{k}+\left[\begin{array}{c} {\mathrm{SL}}_{t,t+1}\cdot \cos\left(\phi \right)\\ {\mathrm{SL}}_{t,t+1}\cdot \sin\left(\phi \right) \end{array}\right]\right)∥}_{\sum_{k+1}^{PDR}}^{2} \end{array}$$

**GNSS–SPP and PPK factor:** GNSS–SPP factor is obtained by the smartphone raw GNSS observation with SPP solution.

(24) $${∥e_{k+1}^{GNSS}∥}_{\sum_{k+1}^{GNSS}}^{2}={∥X_{k+1}-GNSS_{k+1}∥}_{\sum_{k+1}^{GNSS}}^{2}$$

Therefore,

(25) $$\hat{X}=\arg\min\left(\sum_{i=1}^{k}\left[{∥e_{i+1}^{PDR}∥}_{\sum_{i+1}^{PDR}}^{2}+{∥e_{i+1}^{GNSS}∥}_{\sum_{i+1}^{GNSS}}^{2}\right]\right)$$

The GNSS–PPK factor is created by replacing the GNSS with GNSS–PPK data.

We used the Georgia Tech Smoothing and Mapping (GTSAM) library [36] with the Levenberg–Marquardt (LM) optimizer [37].

## 3. Experimental Evaluation

### 3.1. Experimental Description

**Figure 4:**
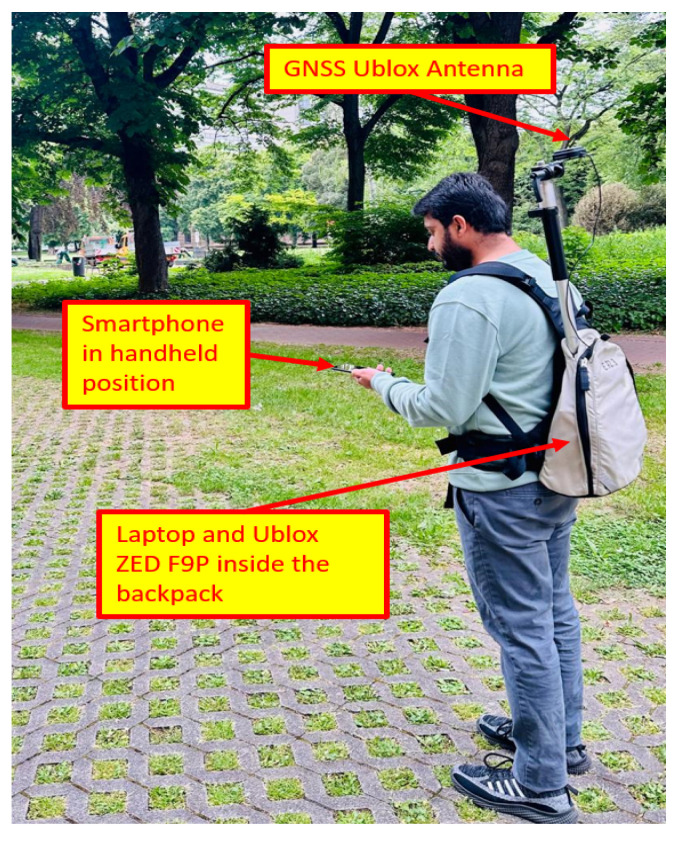
Data collection setup.

**Figure 5:**
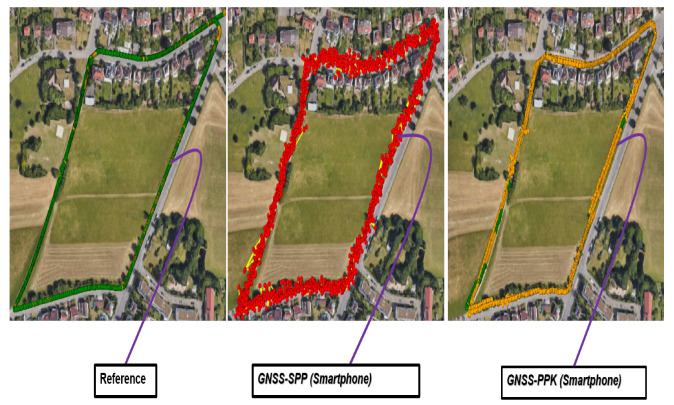
Difference between the ground truth, SPP-GNSS, and PPK-GNSS data.

The experiment aimed to evaluate the reliability of the Google Pixel 4 smartphone’s GNSS for high-precision localization by comparing its accuracy with that of a high-accuracy Ublox ZED F9P RTK GNSS receiver (Thalwil, Switzerland), which served as the ground truth. The Ublox antenna was placed on top of the pedestrian’s head with a rod on the backpack, and the smartphone was held in a handheld position by the pedestrian, as shown in Figure 4. The experiment was conducted in Korntal Münchingen, Stuttgart, Germany. A closed path of around 920 m, characterized by a partially open sky with trees and houses, was chosen for data collection, which can be seen in Figure 5.The data were collected at a normal walking speed by completing two laps (1.8 km) on the pre-defined path in approximately 27 min. A Google Pixel 4 smartphone was used to collect the smartphone’s built-in GNSS and inertial sensor measurements. For ground truth, a Ublox ZED F9P RTK GNSS receiver was used, which is capable of providing centimeter-level accuracy. The pedestrian carried the smartphone in front of his chest, while the Ublox’s ANN-MB-00 GNSS antenna was mounted on a backpack, as can be seen in Figure 4.

The raw GNSS observations from the Android smartphone were logged using the GNSSLogger app [38], which conveniently allows data to be recorded directly in the RINEX format. For processing these observations with the PPK GNSS technique, we utilized the open-source RTKLIB Explorer2.4.3 software [39]. The RINEX observation file obtained from the smartphone contained numerous errors inherent to raw data, including satellite clock errors, inaccuracies in the satellite orbit and position, ionospheric delays, wet and dry tropospheric delays, receiver clock offsets, as well as multipath effects and non-line-of-sight (NLOS) errors caused by obstructions such as trees and nearby buildings.

To mitigate these errors, we employed the PPK technique, which applies corrections to the noisy observation file using data from a nearby base station. The RTKLIB software also offers built-in corrections for ionospheric and tropospheric delays, satellite clock errors, and other necessary adjustments. In this study, we corrected the RINEX observation file by selecting signals from multiple GNSS constellations, including GPS, GLONASS, Galileo, and BeiDou. However, we disabled the SBAS correction system because our focus was on evaluating the fusion of PPK-based GNSS data with Pedestrian Dead Reckoning (PDR) rather than on SBAS corrections.

For satellite clocks and orbits, we relied on broadcast ephemerides rather than precise clock products, as our study did not focus on Precise Point Positioning (PPP). Ionospheric errors were corrected using the broadcast ionospheric model, with the impact of these errors further reduced by the use of dual-frequency observations, which are less susceptible to ionospheric disturbances compared with single-frequency data. For tropospheric corrections, we used an estimated Zenith Tropospheric Delay (ZTD) model, which is given by

$$\mathrm{ZTD}=0.0022768\cdot \frac{P}{f(\phi ,H)}+\mathrm{ZWD}$$

where *P* is the atmospheric pressure at the receiver’s location (in hPa). f(ϕ,H) is a function of the latitude ϕ and height *H* above sea level, accounting for the elevation and atmospheric conditions.

Carrier phase ambiguity resolution was implemented only for the GPS constellation, with ambiguity resolution disabled for other constellations. This selective ambiguity resolution allowed us to achieve integer-cycle resolution for GPS signals. These parameters, which are preconfigured in the RTKLIB software, were used to correct the observations. Additionally, we applied both forward and backward filtering, resulting in a combined filter approach that enhanced the accuracy and reliability of the corrections applied to the GNSS data. Moreover, smartphone low-cost chip size GNSS chip receivers and antennas are particularly susceptible to user-side errors such as clock biases, hardware inaccuracies, and antenna offsets [5,35]. Although we are aware of these issues, they were not addressed in this paper.

**Figure 6:**
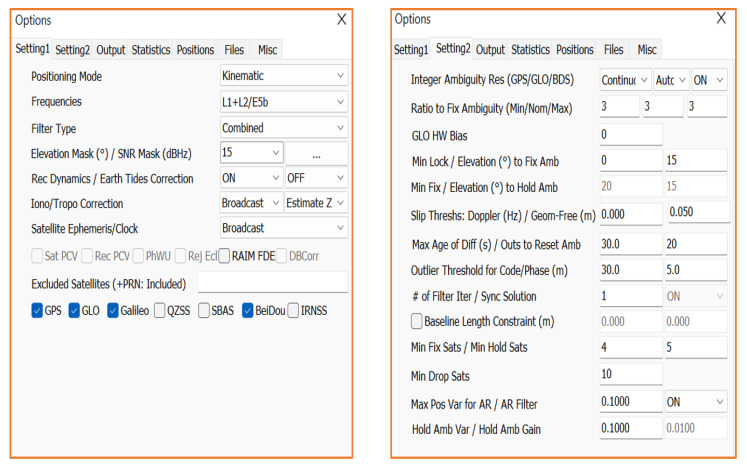
RTKLIB settings.

For processing the data, we utilized the L1, L2, and E5b frequencies. A 15-degree elevation mask was applied, with receiver dynamics enabled and tidal corrections disabled. For integer ambiguity resolution, we employed specific strategies for different satellite systems: continuous-resolution was used for GPS, while auto-calculation was selected for GLONASS (GLO) and BeiDou (BDS) was kept ON, as can be seen in Figure 6.

These settings were chosen after testing various configurations, as they provided a more reliable fixed solution. Specifically, we set the ratio for fixing ambiguity at 3. The elevation mask for fixing ambiguities was also set at 15 degrees, with the minimum fix elevation and minimum elevation to hold ambiguity configured at 20 degrees and 15 degrees, respectively. Additionally, an outlier threshold of 5 m was established for carrier phase measurements.

The base station, located approximately 17 km from the experimental site, provided Radio Technical Commission for Maritime Services (RTCM) correction data through the Satellite Positioning Service (SAPOS) in Baden-Württemberg, Germany. The RTKLIB parameters that we used in the software settings described above for post-processing the smartphone raw GNSS observations are listed in Table 1. With these parameters, we achieved almost 11% fixed and 89% float solutions for smartphone GNSS. In contrast, through Ublox ZED F9P, the raw GNSS data were logged in its .ubx (Ublox) format. Therefore, we first converted that into RINEX format using the RTKConv tool from the RTKLIB. By processing this RINEX data with RTCM correction data of the same base station and with the same RTKLIB parameters as listed in Table 1, we yielded a fixed solution rate of 96.9%. The difference between the implementation of PPK on the datasets from Ublox and smartphone GNSS can be further seen in Figure 5. This high fixed solution rate supports the use of the Ublox receiver as ground truth, demonstrating its reliability and efficiency compared with the smartphone GNSS.

**Accurate synchronization of ground truth and smartphone data:** The data from both devices were timely synchronized by maintaining the sampling frequency of both devices. Moreover, while the Ublox receiver and the smartphone were kept approximately one meter apart during data collection, the resulting positional offset was considered but not specifically corrected in this study. This choice was made to maintain a straightforward setup, with our primary focus on the fusion strategy between PDR and GNSS using the PPK technique.

**KF and FGO parameters:** The efficacy of the FGO and KF fusion algorithms hinges on their adept utilization of parameter covariance settings. Throughout this study, we preserved uniform parameter settings for both fusion techniques. Regarding the KF, we set the KF parameters as follows when we parametrize it in our case:

$$P=\left[\begin{array}{cc} 10 & 0\\ 0 & 10 \end{array}\right],\;Q_{k}=\left[\begin{array}{cc} 1 & 0\\ 0 & 1 \end{array}\right],\;R=\left[\begin{array}{cc} 2 & 0\\ 0 & 2 \end{array}\right]$$

where *P* is the initial state covariance matrix, $Q_{k}$ is the process noise covariance matrix, and *R* is the measurement noise covariance matrix.

In the domain of FGO, our approach involves two factors: PDR and GNSS. It is important to recognize that both of these factors come with uncertainties and noise. We have used GTSAM’s odometry and prior noise model classes to handle these uncertainties. We set the same values for both KF and FGO.

odometryNoise=noiseModel.Diagonal.Sigmas([2;2;0.1])

priorNoise=noiseModel.Diagonal.Sigmas([1.4;1.4;0.1])

### 3.2. Results

#### 3.2.1. Position Error

**Figure 7:**
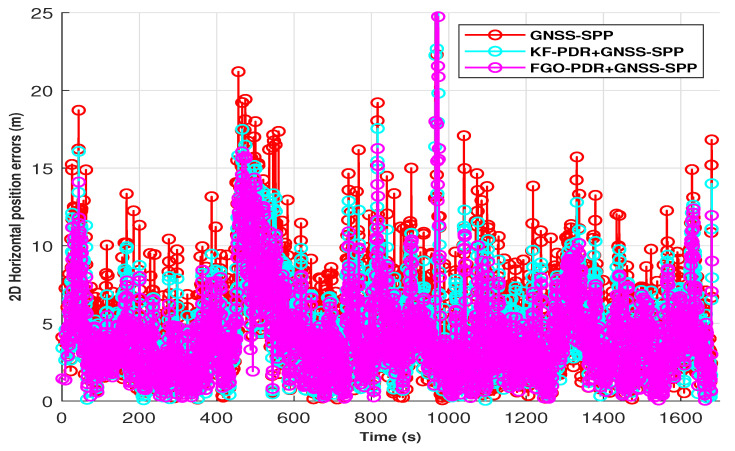
Two-dimensional horizontal positioning errors in PDR+GNSS–SPP fusion architecture.

**Figure 8:**
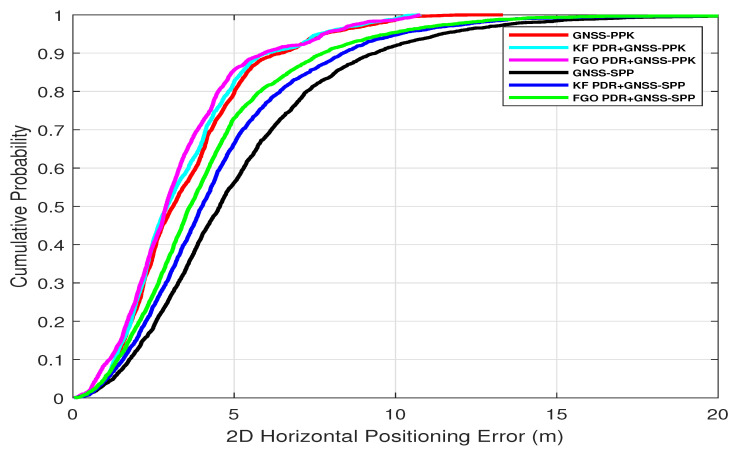
Combined overall CDF from both fusion architectures using FGO and KF.

**Figure 9:**
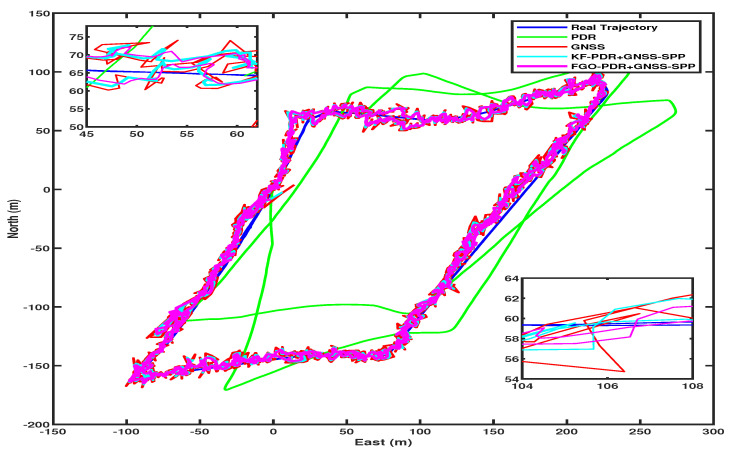
Comparison of trajectories in PDR+GNSS–SPP fusion architectures.

In the semi-open sky condition, the SPP solution of smartphone GNSS was affected by the influence of trees and houses. This effect can be seen in Figure 7in the form of horizontal position error peaks shown in red. Therefore, it can also be seen that the fusion of this GNSS–SPP with PDR from both KF and FGO is producing better results as compared with only GNSS–SPP by mitigating the peak errors of GNSS–SPP. However, we achieved lower positioning errors with FGO compared with KF. This is because of FGO’s iterative optimization of historical measurements, which results in improved accuracy, while KF relies on current and last measurements only. We computed the Cumulative Distribution Function (CDF) of these positioning errors obtained by FGO, KF, and GNSS–SPP, as shown in Figure 8, and from this, it is clear that FGO-based fusion provides overall better accuracy and precision than KF. It can also be observed from the fused trajectories presented in Figure 9 that FGO is more robust than KF.

**Figure 10:**
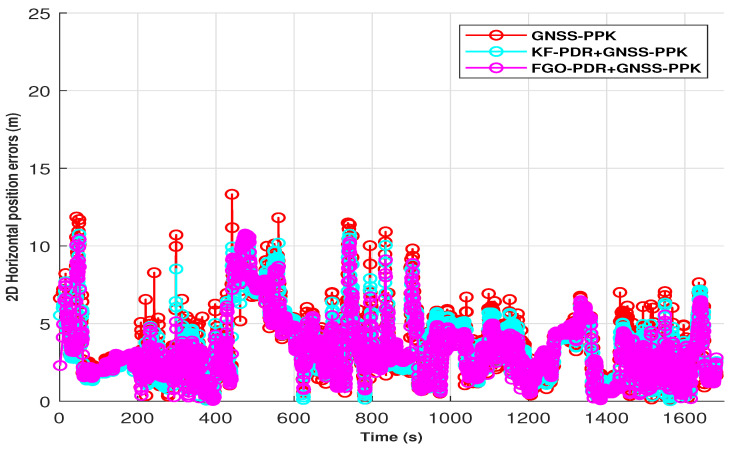
Two-dimensional horizontal positioning errors in PDR+GNSS–PPK fusion architecture.

**Figure 11:**
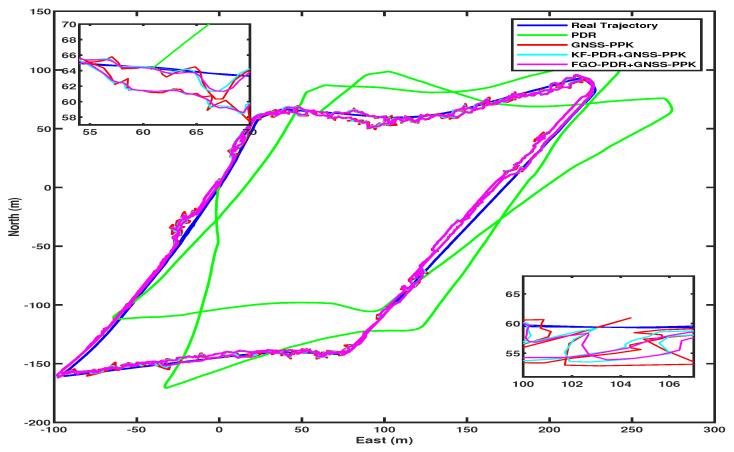
Comparison of trajectories in PDR+GNSS–PPK fusion architecture.

For PPK, we enhanced smartphone raw GNSS measurements using RTCM messages from a base station, processed with RTKLIB software using various parameters shown in Table 1. Consequentially, GNSS error peaks were lower than SPP, as shown in Figure 10 by the red color. Fusing these GNSS–PPK with PDR produced better, smoother, and more precise output than PDR+GNSS–SPP by both fusion algorithms, KF and FGO. However, again, FGO produced better output than KF and only GNSS–PPK, which can be clearly seen from the CDF of horizontal position errors in Figure 8. The trajectories from FGO, KF, and GNSS–PPK can be seen in Figure 11.

#### 3.2.2. Computational Load

**Figure 12:**
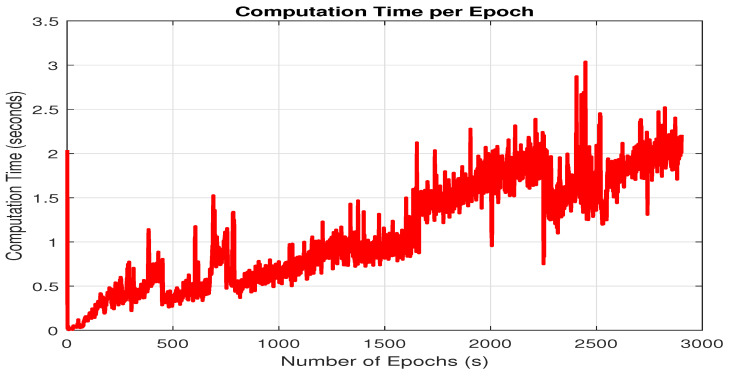
Computational time consumed by FGO-based PDR+GNSS–PPK fusion architecture.

**Figure 13:**
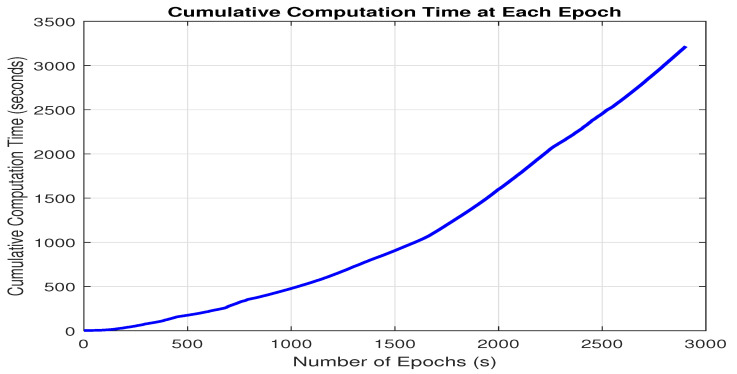
Cumulative computational time consumed by FGO-based PDR+GNSS–PPK fusion.

FGO-based PDR+GNSS–PPK has demonstrated superior positional accuracy compared with other methods discussed in this study. However, it is crucial to assess the computational demands of the FGO approach due to its iterative optimization process. FGO performs optimization at each epoch, incorporating the entire dataset, which can significantly impact computational efficiency. To evaluate the computational load, we analyzed the performance of FGO at each epoch, measuring the amount of computational power utilized. This analysis includes both the per-epoch computational load and the cumulative computational load, which accounts for the computational resources used across all previous epochs. The results of this evaluation are illustrated in Figure 12 and Figure 13. It can be noted that Figure 12 has some peaks at several epochs. This is because of unexpected variations in the data, such as noise or outliers, which require additional computations. The LM optimizer may also encounter conditions at specific epochs that demand more iterations or complex calculations to converge. Additionally, system-related overheads, like memory management or concurrent processes, could contribute to these temporary spikes. The slight increase in computational load per epoch might result from subtle changes in system state, solver efficiency, or the gradual accumulation of numerical errors over time.

Figure 13 shows that the computational load of FGO increases with each new epoch. This happens because FGO needs to optimize all factors from scratch at each epoch, which requires more computing power. By the end, the computational load has grown significantly, reaching around 5320 s. This increase is due to FGO’s iterative process, which re-optimizes the entire dataset at every epoch. This repeated optimization and consideration of all factors makes FGO quite resource-intensive, which makes it less practical for real-time applications where efficiency is important.

### 3.3. Discussion

**Figure 14:**
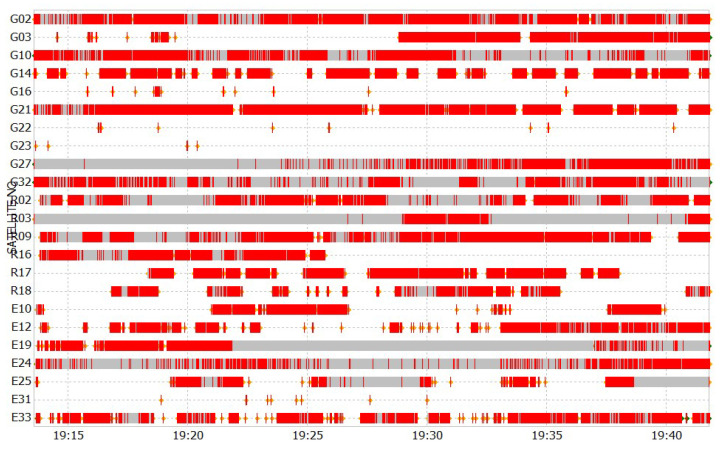
Google Pixel 4 raw GNSS observartions.

To characterize the positional performance of PDR+GNSS–PPK with FGO, we computed the mean, median, and standard deviation (STD). Subsequently, we compared its mean % improvement with PDR+GNSS–SPP, GNSS–SPP, GNSS–PPK, KF–PDR+GNSS–SPP, and KF–PDR+GNSS–PPK, as detailed in Table 2. FGO with PDR+GNSS–PPK has achieved 22.5% better 2D mean error compared with FGO fusion with PDR+GNSS–SPP, which shows the higher capability of smartphone positioning in partial-open sky conditions, but still, for PPK, we have received positional errors in the range of 2–3 m. This is due to the fact that the fixed ambiguity solution from smartphone raw GNSS observation was very low, which is 11%. This is mainly due to the limitations of the smartphone’s low-quality chip size GNSS receiver, which resulted in continuous cycle slips across all satellites for significant portions of the data and also resulted in hardware clock discontinuities at every epoch, likely caused by the phone’s duty cycling of GNSS tracking during data collection, which can be seen in Figure 14, which shows simultaneous observations from a Google Pixel 4, where the red ticks indicate the flagged cycle slips. With few to no valid carrier phase observations in the degraded files, these solutions often exhibited very large position errors. Similarly, it was highlighted by other studies as well that a smartphone’s GNSS performance is prone to several limitations [5,34,40,41,42,43].

Despite the limited corrections applied to the raw GNSS observations, we observed improved positioning accuracy compared with KF-based fusion. This enhancement is advantageous for various applications. However, FGO’s computational performance is quite high, making it impractical for real-time applications. This limitation arises from FGO’s batch processing mode, which optimizes the entire dataset in each iteration. A potential solution to manage this issue could be the use of a sliding window-based optimization approach. While our offline implementation showcases promising results, that can be useful for some applications. However, further examination is needed to assess its feasibility and practicality in diverse operational scenarios.

## 4. Conclusions and Future Work

Our study implemented GNSS–PPK and its fusion with PDR using FGO under partially open sky conditions, surrounded by homes and trees. We compared its performance with GNSS–SPP fusion with PDR via both FGO and KF, PDR+GNSS–PPK via KF, as well as only GNSS–SPP and GNSS–PPK. From the results obtained, we observe that for enhanced accuracy, the FGO-based PDR+GNSS–PPK offers a superior option for seamless localization. However, the computational load for FGO-based PDR+GNSS–PPK is much higher to be implemented in real-time in smartphones; the computational load at each epoch can be seen in Figure 12, and the overall accumulative computational time consumed can be seen in Figure 13. Moreover, it can be noted that PPK is a post-processed GNSS technique, which makes it unsuitable for real-time applications. Therefore, in the future, we plan to test our system in real-time by setting up our own base station. We believe that establishing a base station for real-time testing is a crucial next step. Consequently, we believe that the following works are essential for future research:

(1) It is essential to explore the balance between computational load and the performance of FGO–PDR/GNSS systems. Implementing a sliding window for optimization could be a promising approach to reduce computational demands while preserving the system’s performance.
(2) We want to assess our fusion architecture’s performance using more datasets in a variety of complex environments, including real-time smartphone locations and deep urban canyons, using sliding window-based FGO to maintain a healthy balance between computational complexity and accuracy.

## Figures and Tables

**Table 1:** RTKLIB parameters.

Positioning Mode	Kinematic
Filter Type	Combined
GNSS Constellations	Disable QZSS, IRNSS, and SBAS
Ionospheric Correction	Broadcast
Tropospheric Correction	Estimate ZTD
Satellite Clock	Broadcast
Integer AR	Continuous for GPS

**Table 2:** Two-dimensional error analysis.

Approach	GNSS Processing Techniques						% FGO Mean Improvement over GNSS and KF
	**PPK**			**SPP**			
	**Mean Error**	**Median Error**	**STD**	**Mean Error**	**Median Error**	**STD**	
GNSS (only)	3.56 m	3.11 m	2.10 m	5.23 m	4.55 m	3.56 m	7.28% (PPK) and 18.5% (SPP)
KF–PDR+GNSS	3.44 m	2.92 m	2.04 m	4.60 m	4.00 m	3.16 m	4.36% (PPK) and 7.60% (SPP)
FGO–PDR+GNSS	3.29 m	2.88 m	2.03 m	4.25 m	3.62 m	3.02 m	as compared with SPP 22.5%

## Data Availability

The data that support the findings of this study are available from the corresponding author, upon reasonable request.

## Abbreviations

The following abbreviations are used in this manuscript:

FGO	Factor Graph Optimization
GNSS	Global Navigation Satellite Systems
IMU	Inertial Measurement Unit
KF	Kalman Filter
NLOS	Non-Line-of-Sight
PNT	Positioning Navigation and Timing
PDR	Pedestrian Dead Reckoning
SPP	Single-Point Positioning
PPK	Post-Processing Kinematics
